# Involved-field irradiation or elective-nodal irradiation in neoadjuvant chemo-radiotherapy for locally-advanced esophageal cancer: comprehensive analysis for dosimetry, treatment-related complications, impact on lymphocyte, patterns of failure and survival

**DOI:** 10.3389/fonc.2023.1274924

**Published:** 2023-10-10

**Authors:** Xianyan Chen, Yingjie Zhang, Xiaojuan Zhou, Min Wang, Feifei Na, Lin Zhou, Yong Xu, Bingwen Zou, Jianxin Xue, Yongmei Liu, Youling Gong

**Affiliations:** ^1^ Division of Thoracic Tumor Multidisciplinary Treatment, Cancer Center and State Key Laboratory of Biotherapy, West China Hospital, Sichuan University, Chengdu, China; ^2^ Physics Center, Cancer Center, West China Hospital, Sichuan University, Chengdu, China

**Keywords:** esophageal squamous cell carcinoma, neoadjuvant chemo-radiotherapy, involved-field irradiation, elective nodal irradiation, lymphopenia

## Abstract

**Purpose:**

To compare the differences between involved-field irradiation (IFI) and elective nodal irradiation (ENI) in selecting the optimal target area for neoadjuvant chemoradiotherapy (nCRT) in patients with locally advanced esophageal squamous cell carcinoma (LA-ESCC).

**Materials and methods:**

We retrospectively analyzed 267 patients with LA-ESCC, of whom 165 underwent ENI and 102 underwent IFI. Dosimetry, treatment-related complications, pathological responses, recurrence/metastasis patterns, and survival were compared between the two groups.

**Results:**

The median follow-up duration was 27.9 months. The R0 resection rates in the IFI and ENI groups were 95.1% and 92.7%, respectively (*p*=0.441), while the pathological complete response (pCR) rates were 42.2% and 34.5%, respectively (*p*=0.12). The ENI group received higher radiation doses to the heart (HV_30_:23.9% *vs*. 18%, *p*=0.033) and lungs (LV_30_:7.7% *vs.* 4.9%, *p*<0.001) than the IFI group. Consequently, the ENI group showed a higher incidence of grade 2 or higher radiation pneumonitis (30.3% *vs.* 17.6%, *p*=0.004) and pericardial effusion (26.7% *vs.* 11.8%, *p*=0.021) than the IFI group. Post-operation fistulas were observed in 3 (2.9%) and 17 cases (10.3%) in the IFI and ENI groups, respectively (*p*=0.026). In the multivariate analysis, smoking, positive lymph node involvement (pN+), and anastomotic fistula were independent predictors of overall survival (OS). The pN+ patients exhibited a greater propensity for recurrence compared to pN- patients, especially in the first year of follow-up (6.67% *vs.* 0.56%, *p*=0.003).

**Conclusion:**

The ENI group had a higher incidence of radiation-induced adverse events compared to the IFI group, likely due to the higher radiation doses to normal tissues. Considering the similar disease-free survival (DFS) and OS rates in the two groups, IFI may be suitable for nCRT in patients with LA-ESCC, although further prospective studies are warranted.

## Introduction

Esophageal cancer is the seventh most prevalent malignancy and the sixth leading cause of cancer-related fatalities (1). Despite advances in treatment, the disease is characterized by a high incidence of local and distant recurrence following surgical resection, leading to a dismal 5-year OS rate that rarely exceeds 30% (2). The long-term findings of the landmark CROSS trial established the survival benefits of neoadjuvant radiotherapy combined with surgery for resectable esophageal cancer, setting the standard of care for locally advanced cases (3, 4). However, the optimal radiation field for neoadjuvant radiotherapy remains controversial. Although the CROSS trial assessed the efficacy and recurrence patterns in the involved-field irradiation (IFI) group, it did not compare these parameters with those in the elective nodal irradiation (ENI) group (3). A retrospective study including 118 patients with esophageal squamous cell carcinoma (ESCC) receiving neoadjuvant chemoradiotherapy (nCRT) compared the efficacy and failure patterns between the ENI and non-ENI groups, as both were applicable to most of the population because of the similar prognoses of the two groups. However, considering the higher risk of complications in older patients (>70 years old), the authors recommended IFI only for this subgroup (5).

This study compared the application of ENI and IFI to evaluate the optimal radiation fields in terms of the dosimetric parameters, chemoradiotherapy-related and operational complications, impact on lymphocytes, pathological response, disease-progression patterns, and clinical outcomes.

## Materials and methods

### Patients

Patients with locally advanced esophageal cancer (AJCC Ver. 8, stages II–IVA) were reviewed at the West China Hospital between March 2017 and October 2022. All patients had pathologically confirmed squamous cell carcinoma and underwent nCRT followed by radical esophagectomy. Patients with performance status (PS) ≥ 2, distant metastases, prior chest radiation therapy, or coexisting other malignant tumors were excluded. The clinical stage was assessed by the following examinations, including esophagography, endoscopy, and computed tomography (CT), with some patients undergoing positron emission tomography.

### Neoadjuvant chemo-radiotherapy

All patients received nCRT, with radiotherapy being delivered through intensity-modulated radiotherapy (IMRT). For IFI, the gross tumor volume (GTV) was the sum of the primary lesion (GTVp) and metastatic lymph node volume (GTVn). The lymph node clinical target volume (CTVn) included the GTVn with additional 5-mm expansion (6, 7). The CTV was defined as the sum of CTVn and GTVp plus a longitudinal 3-cm margin along the esophagus and a 5-mm radial margin. The planning target volume (PTV) was defined as the CTV with 5-10 mm expansion (8). Differently from IFI, the CTVn for ENI included both the clinically affected and uninvolved lymph node areas or stations (1/2/4/5/7, 2/4/5/7/8/9, and 4/5/7/8/9/16/17, respectively) for upper, middle, and lower thoracic ESCC (9). For normal tissues, the dose-volume constraints were as follows: to the spinal cord, ≤ 36 Gy; to the heart, V_30_ (volume receiving 30 Gy) ≤ 30%, mean heart dose (MHD) < 30 Gy; and to the whole lung, V_20_ ≤ 20%, V_30_ ≤ 15%, and mean lung dose (MLD) < 20 Gy (10). All patients were treated with paclitaxel in combination with carboplatin. Paclitaxel dose was calculated at 50 mg/m^2^ and carboplatin at an area under the curve of 2, weekly for 5 weeks (10).

### Surgery

Surgery was scheduled 4 to 8 weeks after completion of nCRT. Most of the patients underwent McKeown esophagectomy (240/267, 89.9%). R0 resection was defined as complete resection of the tumor, with no tumor visible under the naked eye or microscope. A microscopic residual tumor (R1) was defined as a tumor located < 1 mm from the proximal, distal, or circumferential resection margins.

### Pathological analysis

Pathological complete response (pCR) was defined as the absence of cancer cells in the primary lesion and regional lymph nodes after neoadjuvant therapy. Based on the degree of response of the primary tumor to treatment, the evaluation protocol for esophageal cancer (modified Ryan scheme for tumor regression score) from the College of American Pathologists classified the tumor regression grade (TRG) into four stages as follows: grade 0, no surviving cancer cells; grade 1, single cells or rare small groups of cancer cells; grade 2, residual cancer with evident tumor regression but more than single cells or rare small groups of cancer cells; and grade 3, extensive residual cancer with no evident tumor regression (11, 12).

### Treatment-related complications

Complications of nCRT included hematological toxicity (anemia, thrombocytopenia, leukopenia, neutropenia, and lymphopenia), radiation pneumonia, radiation esophagitis, and radiation heart disease. Postoperative complications included fistula, esophageal stenosis, pleural effusion, pneumothorax, pulmonary atelectasis, acute respiratory distress syndrome (ARDS), mortality, and readmission within 30 days. We scored the severity of treatment-related complications using the Common Terminology Criteria for Adverse Events (CTCAE) 5.0.

### Definition of endpoint and patterns of failure

The main endpoints were OS, disease-free survival (DFS), complications, pathological response, and failure modes. In-field failure (IFF) was defined as the presence of recurrence or metastasis within the irradiated field area. Out-of-field failure (OFF) was defined as the presence of recurrence or metastasis outside the irradiated field area. Patients in both groups had local recurrences.

### Statistical analysis

The x2 test or Fisher’s exact test were used to compare the differences in the patient and tumor characteristics, toxicity, and first failure between the ENI and IFI groups. Spearman’s correlation analysis was performed to analyze the correlation between two continuous variables. The time between the start of nCRT or surgery and date of death, recurrence, or last follow-up was used to compute the survival outcomes. OS and DFS were calculated using the Kaplan-Meier method and compared using the log-rank test. Cox proportional hazard models were used to conduct both single-factor and multi-factor analyses as well as to compute the hazard ratios (HR) and 95% confidence intervals (CI). Variables with *p <*0.1 in the univariate analysis were entered into the multivariate model. SPSS (version 26.0), R Studio (version 4.2.3), and GraphPad Prism 9 software were used for all analyses. Statistical significance was set at *p* < 0.05.

## Results

### Patient characteristics

A total of 267 patients with esophageal cancer who completed preoperative nCRT followed by radical surgery were enrolled in the study. Among these, 102 (38%) were in the IFI group and 165 (62%) were in the ENI group. **Table 1** shows the characteristics of the 267 patients. The median numbers of lymph node dissections were 25 (range:15–46) and 24 (range:16-43) in the IFI and ENI groups, respectively. The R0 resection rates in the IFI and ENI groups were 95.1% and 92.7%, respectively. Age sex, PS, smoking, tumor location, length, and stage, radiation dose, and R0 resection were not significantly different between the IFI and ENI groups (all *p* > 0.05).

**Table 1:** Demographic and baseline variables and treatment characteristics of the study population.

Variables	Entire dataset (N =267), (%)		*p*-value
	IFI	ENI	
Total	102 (100)	165 (100)	
Age, y			.478
Median, (range)	61(46-74)	62(44-75)	
Sex			.326
Male	86(84)	146(88)	
Female	16(16)	19(12)	
ECOG PS			.499
0	82(80)	138(84)	
1	20(20)	27(16)	
Smoking			.682
Yes	42(41)	63(38)	
No	60(59)	102(62)	
Tumor location			.100
Ut/Mt	59(58)	111(67)	
Lt	43(42)	54(33)	
Clinical T status†			.212
T1+T2	12(12)	12(7)	
T3+T4	90(88)	153(93)	
Clinical N status†			.434
N0+N1	50(49)	89(54)	
N2+N3	52(51)	76(46)	
Clinical Stage†			.758
I+II	22(22)	33(20)	
III+IVA	80(80)	132(80)	
Tumor length, cm			.301
Median, (range)	4(2-8)	4(2-11)	
Prescribed dose, Gy			.219
Median, (range)	41.4(40.0-41.4)	41.4(39.6-41.4)	
No. of dissected lymph nodes			.964
Median, (range)	25(15-46)	24(16-43)	
R0 resection			.441
R0	97(95.1)	153(92.7)	

IFI, involved-field irradiation; ENI, elective-nodal irradiation; ECOG PS, Eastern Cooperative Oncology Group Performance Status; Ut, upper thoracic esophagus; Mt, middle thoracic esophagus; Lt, lower thoracic esophagus;

† American Joint Committee on Cancer staging manual, eighth edition.

### Dosimetric parameters

The radiation doses in the IFI and ENI groups were 40.0–41.4 Gy and 39.6–41.4 Gy in 1.8–2.0 Gy fractions, respectively. **Table 2** presents the cardiac and pulmonary dosimetric parameters of the IFI and ENI groups. Patients who underwent ENI had significantly higher heart V_30_ and lung V_5_, V_10_, V_20_, and V_30_ values than those in the IFI group (*p* < 0.05).

**Table 2:** Comparison of dosimetric parameters between ENI and IFI groups.

Dosimetric variables	Entire dataset (N =267)		*p*-value
	IFI	ENI	
PTV, cm^3^			
Median, IQR	305.7(217.9-420.4)	457.6(321.3-539.5)	<0.001
Heart			
Mean heart dose, Gy	19.8(17.5-24.2)	21.2(18.1-24.3)	.658
HV_5_	95.6(85.6-99)	91.6(53.2-98.0)	.102
HV_10_	83.0(68.5-90.3)	80.3(64.9-89.0)	.278
HV_20_	44.5(35.8-58.0)	51.0(39.8-60.0)	.257
HV_30_	18.0(13.1-30.0)	23.9(16.5-30.9)	.033
Lung			
Mean lung dose, Gy	9.3(7.7-11.2)	10.6(9.0-11.7)	.380
LV_5_	45.7(40.1-52.4)	51.5(45.4-55.7)	.025
LV_10_	32.7(27.9-37.9)	37.0(31.9-40.1)	.042
LV_20_	16.5(11.1-21.4)	19.6(15.7-23.0)	<0.001
LV_30_	4.9(2.0-8.5)	7.7(5.0-11.0)	<0.001

IFI, involved-field irradiation; ENI, elective nodal irradiation; PTV, planning target volume; IQR, interquartile range; Vx, the percentage of lung/heart volume receiving ≥x Gy.

### Treatment-related complications

All serious adverse events that occurred during treatment are summarized in **Table 3**. Hematological toxicity has emerged as the most prevalent complication of radiation therapy. In the IFI and ENI groups, grade 3 or higher leukopenia was observed in 35 (34.3%) and 36 (21.8%) patients, respectively, and neutropenia was observed in 30 (29.4%) and 23(13.9%) (*p* < 0.05) patients, respectively. Grade 2 pericardial effusion (*p*=0.021) and radiation pneumonitis (*p*=0.004) occurred at considerably higher rates in the ENI group than in the IFI group (30.3% *vs.* 17.6% and 26.7% *vs.* 11.8%, respectively). No significant differences in arrhythmia or radiation esophagitis were observed between the two groups. There were 20 postoperative fistulas: three (2.9%) in the IFI group and 17 (10.3%) in the ENI group (*p*=0.026). Within 30 days of surgery, four patients in the IFI group and six in the ENI group were readmitted for anastomotic fistula (seven cases), respiratory failure (two cases), and wound infection (one case). There were two deaths in each group within 30 days after surgery: two from hemorrhage and two from severe pneumonia. Intraoperative bleeding, surgery duration, ARDS, pleural effusion, pneumothorax, atelectasis, and esophageal stenosis were not significantly different between the two groups (all *p* > 0.05).

**Table 3:** Adverse events analysis based on neoadjuvant chemoradiotherapy and surgery.

	IFI (n=102)	ENI (n=165)	*p*-value
	n (%)	n (%)	
nCRT-related events			
Anemia†	2(2)	2(1.2)	.625
Thrombocytopenia†	2(2)	4(2.4)	.804
Leukopenia§	35(34.3)	36(21.8)	.025
Neutropenia§	30(29.4)	23(13.9)	.002
G4 Lymphopenia	23(21.5)	41(25.2)	.406
ΔALC, 10^9^/L, IQR	0.79(0.51-1.06)	1.01(0.62-1.30)	.019
Pericardial effusion*	18(17.6)	50(30.3)	.021
Arrhythmia*	12(11.8)	24(14.6)	.583
Radiation pneumonitis*	12(11.8)	44(26.7)	.004
Radiation esophagitis*	13(12.7)	19(11.5)	.102
Surgery-related events			
Bleeding, ml, IQR	50(50-100)	50(50-100)	.338
Surgery duration, min, IQR	270(240-296)	275(240-332)	.168
Fistula†	3(2.9)	17(10.3)	.026
ARDS†	1(1)	2(1.2)	.861
Pleural effusion*	11(10.8)	16(9.7)	.775
Pneumothorax¶	26(25.5)	43(26.1)	.918
Atelectasis¶	18(17.6)	38(23)	.294
Anastomotic stenosis¶	38(37.3)	54(32.7)	.449
30 days re-hospitalization	4(3.9)	6(3.6)	.905
Death after 30 days	2(2)	2(1.2)	.625

IFI, involved-field irradiation; ENI, elective nodal irradiation; nCRT, neoadjuvant chemoradiotherapy; ALC, absolute lymphocyte count; G4, grade 4; ΔALC= [mean ALC pre-nCRT] – [mean ALC post-nCRT]; IQR, interquartile range; ARDS, acute respiratory distress syndrome. †, Events of grade ≥3 according to CTCAE 5.0. §, Events of grade ≥3 with fever or grade 4 according to CTCAE 5.0. *, Events of grade ≥2 according to CTCAE 5.0; ¶, Events of any grade according to CTCAE 5.0.

### Impact on lymphocytes

**Figure 1:**
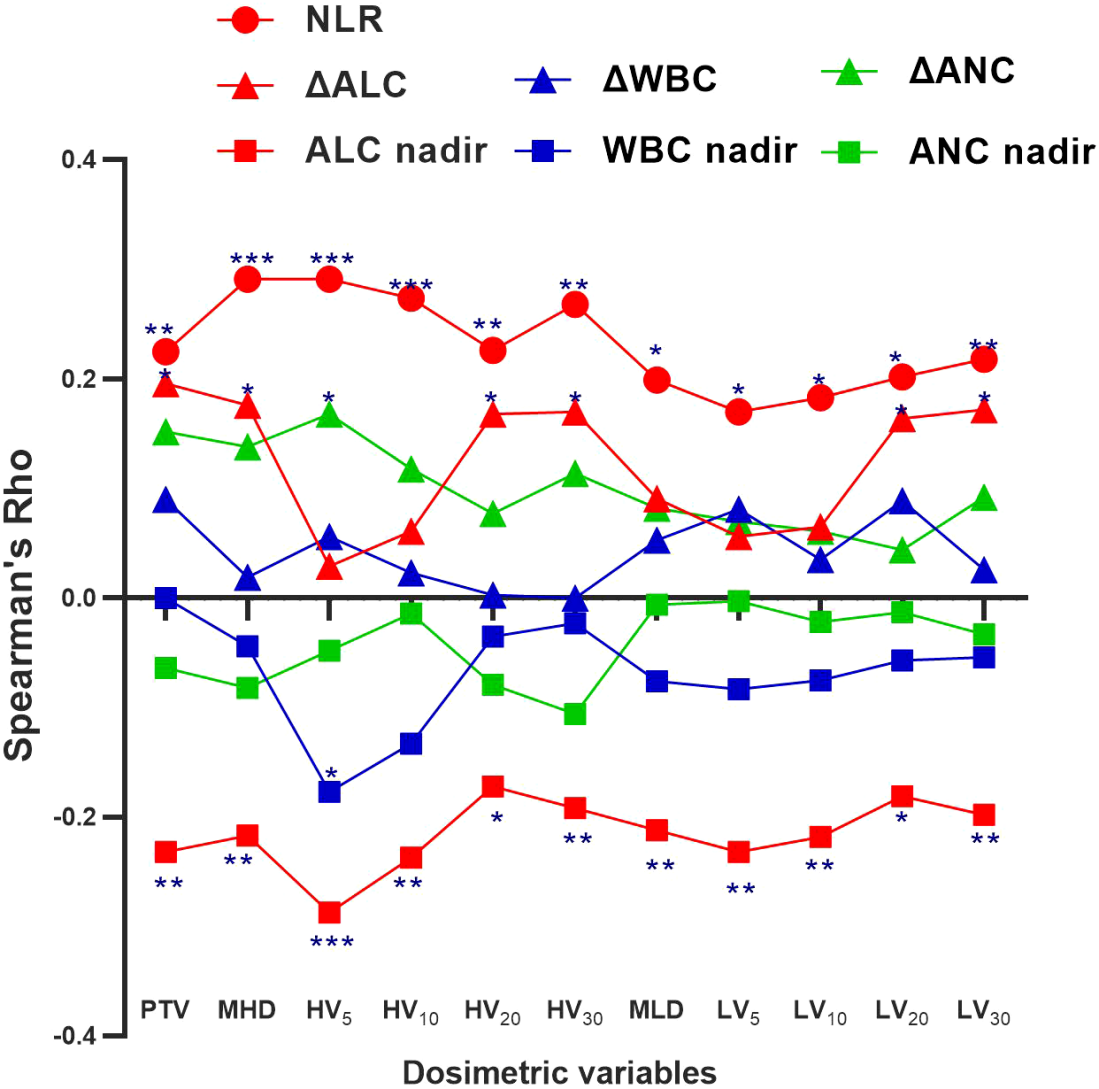
Spearman correlation coefficients (Rho) between the cardio-pulmonary dosimetric parameters and absolute lymphocyte count (ALC) nadir, neutrophil-lymphocyte ratio (NLR), and ΔALC, white blood cells (WBC) nadir, ΔWBC, absolute neutrophil count (ANC) nadir, and ΔANC. Significance indicated at ***p <.001, **p <.01, and *p <.05.

**Table 4** shows that higher cardiopulmonary radiation doses (MHD, HV_10-30_, and LV_5-30_) were strongly associated with grade 4 (G4) lymphopenia (*p*<0.05). **Table 3** demonstrates that the Δabsolute lymphocyte count (ΔALC= [mean ALC pre-nCRT] – [mean ALC post-nCRT]) was significantly lower in the IFI group than in the ENI group (0.79 *vs.* 1.01, *p*=0.019). Spearman’s correlation graph was plotted (including the leucocyte, neutrophil granulocyte, and lymphocyte) with the cardiopulmonary dosimetric parameters (**Figure 1**). A larger PTV and higher cardio-pulmonary doses (MHD, HV_20_, HV_30_, LV_20_, LV_30_) were strongly associated with ΔALC, while all cardio-pulmonary doses were highly correlated with the ALC nadir and neutrophil-lymphocyte ratio (NLR) (each *p* < 0.05). Only HV_5_ shows a significant correlation with leucocyte/neutrophil granulocyte (*p*<0.05), while other cardio-pulmonary parameters had not shown the significant correlation.

**Table 4:** Logistic regression analysis of grade 4 (G4) lymphopenia during nCRT.

Variables	Univariate analysis	
	OR (95% CI)	*p*-value
ENI *vs*. IFI	0.754(0.387-1.469)	.406
PTV, cm^3^	1.003(1.001-1.005)	.004
Heart		
MHD, Gy	1.099(1.023-1.180)	.010
HV_5_	1.025(0.998-1.052)	.068
HV_10_	1.025(1.002-1.049)	.033
HV_20_	1.026(1.003-1.048)	.024
HV_30_	1.040(1.008-1.074)	.014
Lung		
MLD, Gy	1.010(0.976-1.045)	.574
LV_5_	1.045(1.005-1.085)	.026
LV_10_	1.048(1.002-1.095)	.039
LV_20_	1.080(1.017-1.147)	.012
LV_30_	1.094(1.013-1.181)	.022

PTV, planning target volume; ENI, elective-nodal irradiation; IFI, involved-field irradiation; nCRT, neoadjuvant chemo-radiotherapy; OR, odds ratio; CI, confidence interval; MHD, mean heart dose; MLD, mean lung dose; Vx, the percentage of lung/heart volume receiving ≥x Gy.

### Pathological response

**Table 5** summarizes the postoperative pathological responses and disease stages. The tumor regression grades in the IFI and ENI group were TRG0:52 (51%) *vs.* 69 (41.8%), TRG1:15 (14.7%) *vs.* 31 (18.8%), TRG2: 33 (32.4%) *vs.* 56 (33.95%), and TRG3:2 (2%) *vs.* 9 (5.5%), respectively. The rates of pCR, ypT0, and ypN0 in the IFI and ENI groups were 43 (42.2%) *vs.* 57 (34.5%), 52 (51%) *vs.* 71 (43%), and 70 (68.6%) *vs.* 107 (64.8%), respectively; however, the differences between the two groups were not statistically significant (*p*>0.05).

**Table 5:** Distribution of pathologic response and stage after surgery.

	IFI (n=102)	ENI (n=165)	*p*-value
	n (%)	n (%)	
Tumor regression grade			
0	52(51)	69(41.8)	.144
1	15(14.7)	31(18.8)	.391
2	33(32.4)	56(33.9)	.789
3	2(2)	9(5.5)	.163
Pathologic stage†			
I	58(56.9)	84(50.9)	.343
II	12(11.8)	22(13.3)	.709
IIIA	14(13.7)	22(13.3)	.927
IIIB	17(16.7)	33(20)	.498
IVA	1(1)	4(2.4)	.398
pCR	43(42.2)	57(34.5)	.212
ypT0	52(51)	71(43)	.155
ypN0	70(68.6)	107(64.8)	.526

IFI, involved-field irradiation; ENI, elective nodal irradiation; pCR, pathologic complete response.

† American Joint Committee on Cancer staging manual, eighth edition.

### Patterns of failure

**Table 6** summarizes the sites of the first treatment failure. Distant metastases (DM) were most common in 35 cases (13.1%) at the following sites: lung (21, 7.8%), bone (five, 1.9%), adrenal gland (four, 1.5%), liver (three, 1.1%), and brain (two, 0.7%). All in-field failure (ALLIFF) and all out-of-field failure (ALLOFF) were observed in 13 (4.9%) and 16 patients (6%), respectively. The probability of metastasis was significantly lower in the ENI group (one [0.6%]) than in the IFI group (six [5.9%]) in cases of isolated OFF (*p*=0.009). Although there was no significant difference between the two groups in the ALLOFF situation, the ENI group showed a lower ALLOFF rate than the IFI group (seven [4.2%] *vs.* nine [8.8%]).

**Table 6:** Sites of the first treatment failure between IFI and ENI groups.

	IFI (n=102)	ENI (n=165)	*p*-value
First failure-no. of patients (%)			
IFF alone	3(2.9)	6(3.6)	.760
OFF alone	6(5.9)	1(0.6)	.009
DM alone	11(10.8)	16(9.7)	.775
IFF+OFF	0(0)	1(0.6)	.431
IFF+DM	0(0)	0(0)	–
OFF+DM	3(2.9)	2(1.2)	.311
IFF+OFF+DM	0(0)	3(1.8)	.171
All IFF	3(2.9)	10(6.1)	.250
All OFF	9(8.8)	7(4.2)	.125
All DM	14(13.7)	21(12.7)	.814

FF, in-field failure; OFF, out-of-field failure; DM, distant metastasis; ALL IFF, all in-field failure; ALL OFF, all out-of-field failure; ALL DM, all distant metastasis.

### Overall survival and prognostic factors

**Figure 2:**
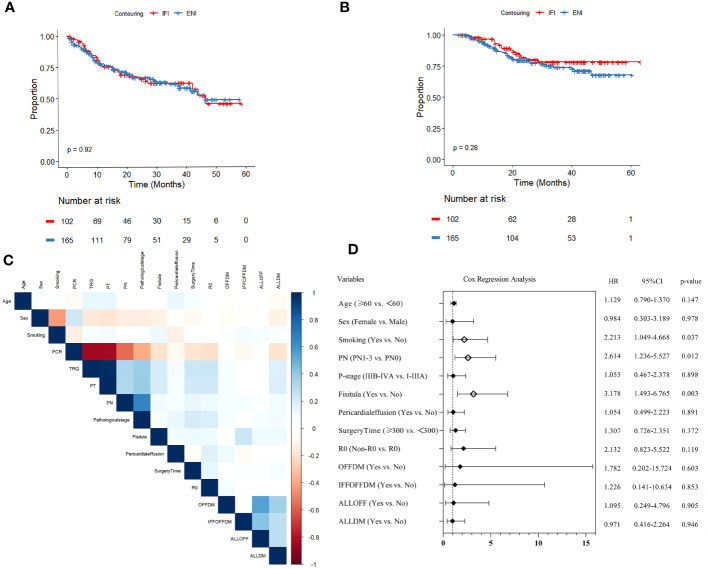
**(A)** Disease-free survival (DFS) analysis and **(B)** overall survival (OS) analysis of elective-nodal irradiation (ENI) and involved-field irradiation (IFI). **(C)** Heatmap of covariance test for variables with *p*<0.1 in univariate analysis. **(D)** Forest plot of Cox multivariable regression analysis for OS.

The median follow-up time for all patients was 27.9 months (range: 7.9–63.3). The 3-year OS and 3-year DFS were (78.2% *vs.* 74.0%) and (62.0% *vs.* 61.5%) for the IFI and ENI groups (**Figures 2A, B**), respectively, but there was no statistical difference (*p*>0.05).

In univariate Cox regression analysis, age, sex, smoking, pCR, TRG, pathological T status (PT), pathological N status (PN), pathological stage, R0, duration of surgery, fistula, pericardial effusion, OFFDM, IFFOFFDM, ALLOFF, and ALLDM were associated with OS (all *p*<0.1). Subsequently, the variables for covariance mentioned above were analyzed, and pCR, TRG, and PT showed strong correlation (r>0.8); therefore, they were excluded from the final multivariate model (**Figure 2C**).

The results of the multivariate Cox regression analysis are presented as forest plots (**Figure 2D**). Ultimately, smoking (HR=2.213, 95% CI:1.049–4.668, *p*=0.037), pN+ (HR=2.614, 95% CI: 1.236–5.527, *p*=0.012), and fistula (HR=3.178, 95% CI: 1.493–6.765, *p*=0.003) were found to be significantly associated with OS.

**Figure 3:**
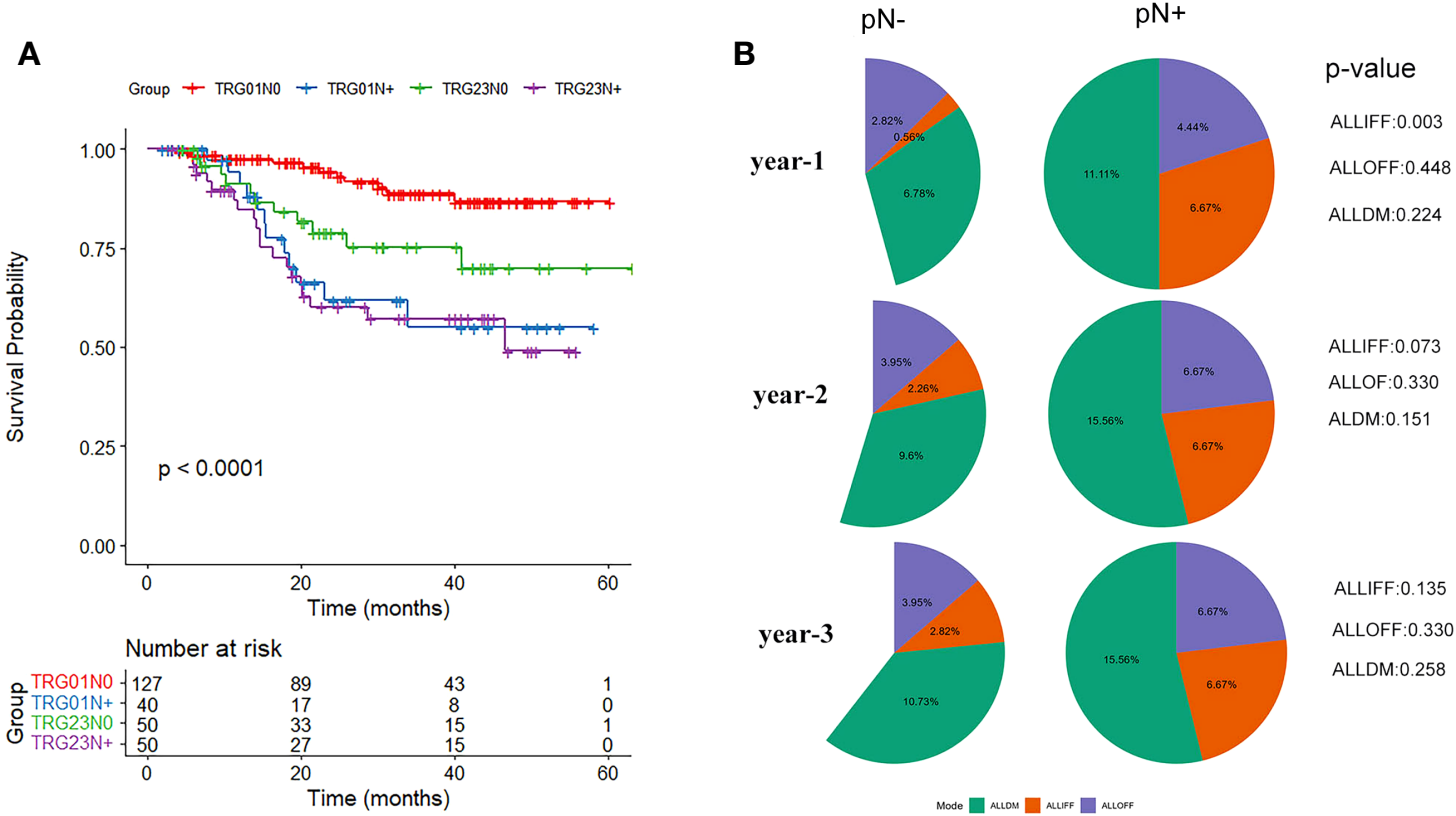
**(A)** Survival analysis curve of tumor regression grade (TRG) combined with pathological lymph node status (N0/N+). **(B)** Differences in pathological lymph node status (pN-/pN+) regarding recurrence and metastasis patterns of all in-field failure (ALLIFF), all out-of-field failure (ALLOFF), and all distant metastases (ALLDM).

Similar prognoses were recorded for TRG0 *vs.* TRG1 (*p*=0.127) and TRG2 *vs.* TRG3 (*p*=0.064), while TRG2/3 had a considerably worse prognosis than TRG0/1 (*p*=0.001). Further analysis was performed based on the combination of TRG and pN0/pN+ status (**Figure 3A**). The results showed that TRG0-1/pN+ patients had a significantly worse prognosis than TRG0-1/pN0 patients (*p*<0.001), while similar survival rates were observed in the other groups (TRG0-1/pN+ *vs.* TRG2-3/pN+, *p*=0.694; TRG0-1/pN+ *vs.* TRG2-3/pN0, *p*=0.234; TRG2-3/pN+ *vs.* TRG2-3/pN0, *p*=0.098).

The differences in the three disease progression patterns of ALLIFF, ALLOFF, and ALLDM between the pN-/pN+ groups are summarized in the follow-up years (**Figure 3B**). The risk of ALLIFF was greater in the pN+ group than in the pN- group (but only in the first year, *p*=0.003). This difference gradually diminished over the next two/three years. Although there was no significant difference in the overall pattern of recurrence between the pN-/pN+ groups, the pN+ group showed a higher tendency of recurrence (25.6% *vs.* 16.4%, *p*=0.074).

## Discussion

ESCC is one of the most prevalent cancers in Asian countries and is typically locally advanced or advanced when first diagnosed and has a high fatality rate (13). In patients with LA-ESCC, the combined use of nCRT and surgery has a considerable survival advantage over surgery alone. The CROSS trial and NEOCRTEC 5010 study laid the foundation for nCRT plus surgery as the standard of care for this patient population (3, 14–16). A large sample study based on the National Cancer Database showed that in neoadjuvant radiotherapy, the pCR and OS did not differ between the three higher radiation doses [39.6-44.9 *vs.* 45-49.9 Gy *vs.* 50 Gy; pCR (*p* = 0.1) *vs.* OS (*p* = 0.097)] (17). While higher radiation doses could increase toxicity, all patients in this study had radiation doses of 39.6-41.4 Gy. However, the current guidelines do not provide explicit recommendations on the scope of target outlining for neoadjuvant RT. Based on the similar values of OS and DFS obtained in our study, the IFI technique might effectively decrease the radiation dose to normal tissues and consequently reduce the treatment-related adverse effects compared to ENI.

ESCC is more likely to metastasize through the esophageal axial lymphatics to multilevel lymph nodes or lymph nodes far from the primary site because of extensive longitudinal lymphatic connections within the esophageal wall (18, 19). The theoretical rationale for ENI is its ability to control lymph node micrometastases and potentially enhance the treatment efficacy by irradiating larger anatomical areas (19). However, the comparative efficacies of IFI and ENI have been found to be inconsistent in many studies on definitive chemoradiotherapy for LA-ESCC. A study conducted at the University of Tokyo Hospital involving 239 cases of esophageal cancer revealed that IFI did not increase the risk of lymph node failure in clinically unaffected nodal stations and demonstrated superior progression-free survival (PFS) and OS compared to the ENI group (20). Similarly, in a study of definitive radiotherapy for locally advanced non-small cell lung cancer (LA-NSCLC), IFI did not increase the incidence of lymph node failure in uninvolved nodal sites but significantly reduced esophageal toxicity (21). Conversely, a retrospective analysis of a larger sample of patients with ESCC favored ENI over IFI in terms of improved OS, with comparable toxicity profiles between the two groups (19). Furthermore, a meta-analysis indicated comparable rates of local control and OS in the ENI and IFI groups; however, the latter exhibited significantly lower incidences of esophageal and pulmonary toxicity (22). Several studies have shown that ENI can reduce local/regional failure; however, its survival benefits remain uncertain (23, 24). One possible explanation could be that the long-term toxicity associated with ENI diminishes the survival advantage (25). However, the comparatively high incidence of distant metastases exerts a more significant negative impact on survival; therefore, the relatively high localized control achieved through ENI may not be converted into a benefit for OS (26).

Few studies have investigated the differences in the efficacies of ENI and IFI in nCRT for LA-ESCC. *Feng* et al. examined the efficacy and failure patterns of ENI and non-ENI in patients with ESCC treated with preoperative irradiation; however, their analysis was not exhaustive (5). While they found that both ENI and IFI were suitable for LA-ESCC owing to similar efficacy outcomes, they recommended IFI for patients over 70 years of age without providing further analysis or explanation for this recommendation. In addition to the aforementioned findings, the present study aimed to address these shortcomings and provide additional insights by conducting new explorations. Our data indicated that patients in the ENI group might have received significantly higher radiation doses to the heart (HV_30_:23.9% *vs*. 18%, *p*=0.033) and lungs (LV_5_-LV_30_, each *p*<0.05) than those in the IFI group. Consequently, the ENI group had notably higher incidence rates of radiation pneumonia (26.7% *vs.* 11.8%, *p*=0.004) and pericardial effusion (30.3% *vs.* 17.6%, *p*=0.021).

Recently, immunotherapy has emerged as a critical therapeutic approach for advanced esophageal cancer, as recommended by the NCCN guidelines (10) In locally advanced solid tumor studies, radiotherapy combined with immunotherapy also appears to overturn the treatment guidelines, such as the KEYNOTE-799 study for LA-NSCLC, which reported a median DFS that far exceeds the current standard PACIFIC treatment paradigm (27, 28). In the field of esophageal cancer, the PALACE-1 study used pembrolizumab in combination with chemoradiotherapy for resectable LA-ESCC and showed a surgery rate of 90% and pCR rate of 55.6% in 20 patients, exceeding the results of the classic CROSS (49.0%) and NEOCRTEC5010 studies (43.2%) (29). Similarly, the NICE study enrolled patients with multisite lymph node metastatic LA-ESCC who were treated with neoadjuvant chemotherapy in combination with camrelizumab immunotherapy and reported a pCR rate of 45.4% (30). Exploratory analyses have highlighted the detrimental effects of excessive irradiation on the heart and large blood vessels, leading to damage to the lymphatic system, which ultimately affects patient survival (31–33). For the first time, our study recorded a significant association between higher cardio-pulmonary irradiation and G4 lymphopenia in the nCRT setting, and the ENI technique was more likely to lead to lymphopenia (*p*=0.019). A recently published study by *Wang* et al. on early-stage NSCLC radiotherapy showed that the estimated dose of radiation to immune cells is critical for treatment outcomes (34). The correlation between lymphocyte count and immunotherapy efficacy has been widely demonstrated in the treatment of a range of solid tumors (35, 36). In fact, the use of IFI to outline the target area provides better protection to the cardiopulmonary system area than ENI, consequently decreasing the incidence of lymphopenia. Thus, the IFI technique theoretically protects the patient’s immune function from irradiation, making it more appropriate to combine PD-1/PD-L1 drugs in the nCRT phase. Several prospective Phase III studies on this treatment are currently ongoing, including NCT05357846, NCT04807673, and NCT05244798. We eagerly await the reports of these studies regarding the clinical benefits, treatment-related toxicity, and other translational findings.

In addition, the present study demonstrated that the pN+ status was strongly associated with a poor prognosis. To further explore this relationship, we compared the recurrence patterns between the pN+/pN- groups, revealing a higher propensity for recurrence in the pN+ group. These findings highlight the importance of performing comprehensive lymph node dissection during surgery to accurately determine the lymph node status. A study by *Samson* et al. showed that compared to patients with esophageal cancer who did not receive adjuvant chemotherapy, those who received adjuvant chemotherapy benefited in terms of OS in all stages of lymph node positivity (37). Therefore, patients with pN+ ESCC may benefit from adjuvant chemotherapy. Recently, the results of the CheckMate-577 study confirmed that adjuvant immunotherapy may improve tumor-free survival in patients with high-risk esophageal cancer who did not achieve pCR after nCRT and R0 surgery, and that adjuvant nivolumab reduced the risk of distant metastases after surgery compared to placebo treatment (29% *vs.* 39%), with a median distant metastasis-free survival of 28.3 months and 17.6 months, respectively (38). Thus, patients with pN+ may benefit from postoperative adjuvant immunotherapy.

Interestingly, our data indicated an increased risk of anastomotic leakage in the ENI group compared to the IFI group according to the surgical procedure, whereas no significant differences were observed in the occurrence of pleural effusion, pneumothorax, esophageal stricture, and other postoperative complications. The larger radiotherapy target area of ENI may cover the anastomotic site in the currently used thoracoscopic approach for esophageal cancer surgery. Radiation exerts negative effects on wound repair through various mechanisms, including vascular system alterations, inflammatory response changes, and cellular function disruption (39, 40). Collagen, a vital matrix protein responsible for the strength and integrity of intestinal wall anastomosis, can be significantly hindered by high radiation doses, thereby affecting anastomotic healing (41, 42). Consequently, considering the range of target areas outlined for nCRT, the IFI may be a more suitable approach in the nCRT settings.

This study has some limitations. First, this study has the inherent limitations associated with both retrospective and observational studies. Future prospective studies with randomized controlled designs will provide stronger evidence in this field. Second, our study focused exclusively on patients with ESCC, which limits the generalizability of our findings to other histological types of esophageal cancer, although almost 95% of them are squamous cell carcinomas in Asian countries. Furthermore, as this was a single-center study, the external validity of our results may be limited. Findings from a single institution may not fully represent diverse patient populations encountered in broader clinical settings. Therefore, we anticipate the emergence of large-scale multicenter prospective studies in the future.

## Conclusion

In conclusion, our study demonstrates that IFI is not inferior to ENI in terms of the pathological response and survival outcomes. The smaller target area of IFI has the potential to reduce cardiopulmonary irradiation, leading to a decrease in treatment-related adverse effects, which theoretically supports the utilization of IFI in nCRT for esophageal cancer. Patients with pN+ disease after nCRT are more likely to experience recurrence and metastasis, which are associated with a poorer prognosis, thus requiring more comprehensive treatment options.

## Data availability statement

The raw data supporting the conclusions of this article will be made available by the authors, without undue reservation.

## Ethics statement

The studies involving humans were approved by the Ethics Committee of West China Hospital (approval number: 2021-1235). The studies were conducted in accordance with the local legislation and institutional requirements. Written informed consent for participation was not required from the participants or the participants’ legal guardians/next of kin in accordance with the national legislation and institutional requirements.

## Author contributions

XC: Data curation, Formal Analysis, Software, Writing – original draft, Writing – review & editing. YZ: Formal Analysis, Methodology, Software, Writing – review & editing. XZ: Data curation, Formal Analysis, Writing – review & editing. MW: Data curation, Formal Analysis, Writing – review & editing. FN: Investigation, Writing – review & editing. LZ: Investigation, Writing – review & editing. YX: Investigation, Writing – review & editing. BZ: Methodology, Writing – review & editing. JX: Methodology, Writing – review & editing. YL: Methodology, Writing – review & editing. YG: Conceptualization, Investigation, Supervision, Writing – review & editing.
